# Heterotaxy syndrome

**DOI:** 10.1590/0100-3984.2017.0063

**Published:** 2018

**Authors:** Lucas Samuel Perinazzo Pauvels, Felipe Welter Langer, Daiane dos Santos, Carlos Jesus Pereira Haygert

**Affiliations:** 1 Department of Radiology and Imaging Diagnosis, Universidade Federal de Santa Maria (UFSM), Santa Maria, RS, Brazil.

*Dear Editor,*

**Figure 1:**
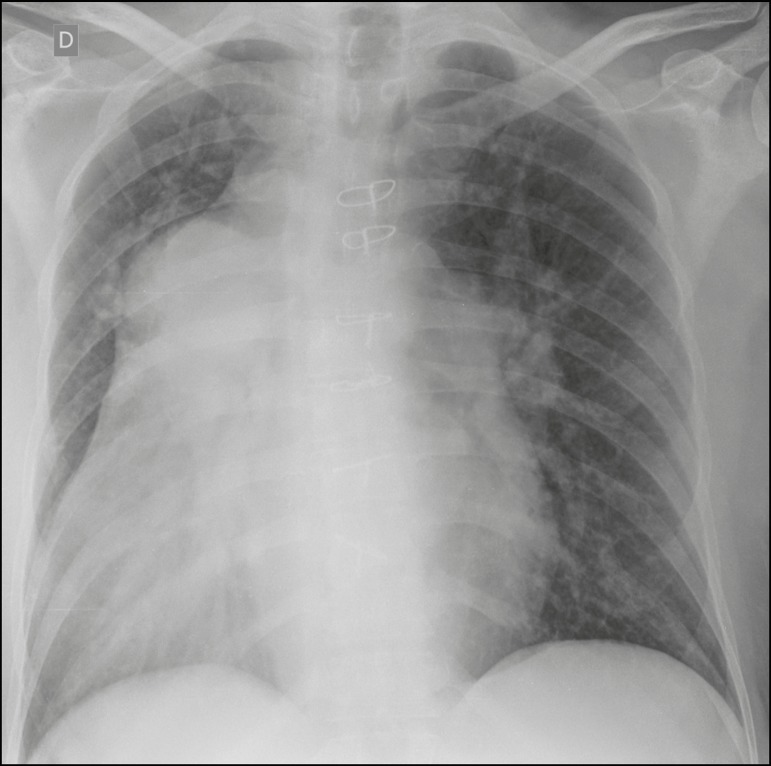
Chest X-ray showing dextrocardia, a widened mediastinum, and enlarged pulmonary arteries suggestive of pulmonary hypertension.

**Figure 2:**
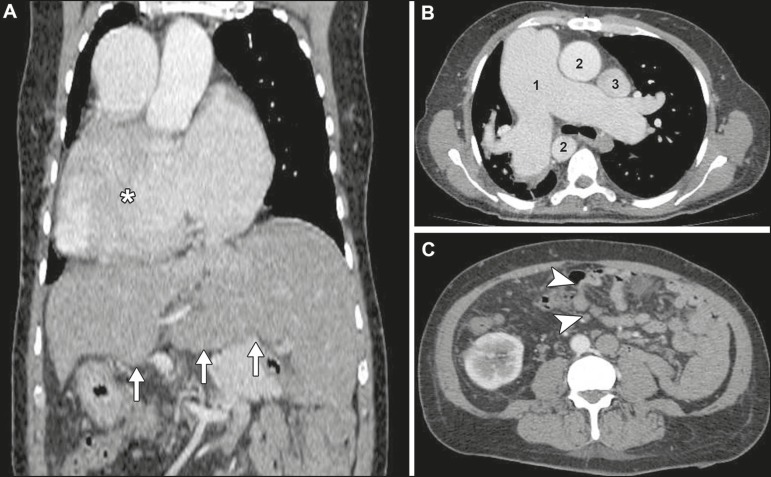
Contrast-enhanced CT scan. A: Coronal thoracoabdominal CT scan showing dextrocardia (asterisk) and a centrally located liver (arrows). B: Axial chest CT demonstrating enlargement of the right-sided pulmonary trunk, which measured 5.8 cm in its largest diameter (1), right-sided aorta (2) and left-sided superior vena cava (3). C: Axial abdominal CT showing abnormal intestinal rotation (arrowheads)—the entire small bowel is positioned to the left of the midline.

A 53-year-old man presented to our neurology department with a progressive, throbbing headache accompanied by focal neurologic deficits. His known medical history included congenital heart disease with dextrocardia, a repaired ventricular septal defect, and a right ventricular-left atrial fistula which had been surgically corrected as well. He also had a long history of unexplained dyspnea. A computed tomography (CT) scan of the head revealed a brain abscess. The patient was admitted to initiate specific therapeutic interventions. However, after admission, he experienced significant worsening of dyspnea, low peripheral oxygen saturation, and cyanosis of the extremities. A chest X-ray showed dextrocardia, an increased cardiothoracic index, and enlargement of the proximal pulmonary arteries (Figure 1), which raised the hypothesis of pulmonary hypertension. Findings on CT angiography, such as severely enlarged pulmonary arteries and filling defects, mainly within the right pulmonary artery, suggested pulmonary hypertension secondary to pulmonary thromboembolism. However, unusual findings were also noted on CT, namely right-sided mediastinum, bilobed right lung, centrally located liver, polysplenia, and abnormal intestinal rotation (Figure 2), all of which were consistent with heterotaxy syndrome (HS). The patient had significant clinical deterioration and died from neurological complications of brain abscess before any curative interventions could be performed.

HS is a rare condition that occurs in approximately 1 in 10,000 live births^(1)^. Patients with HS present with organ arrangement variations other than the typical asymmetry expected in normal anatomy (situs solitus) or its exact mirror image (situs inversus)^(2)^. Normal visceral arrangement depends on a series of intricate processes that take place during early mesoderm development, such as adequate expression and leftward flow of growth signals^(2,3)^. Impairment in any of these factors during organogenesis may lead to abnormal organ positioning and HS.

Patients with HS have historically been classified as having either asplenia (right isomerism) or polysplenia (left isomerism) syndromes^(2-4)^: congenital spleen absence and duplication of right-sided structures characterize the asplenia syndrome, whereas the presence of multiple accessory spleens and duplication of left-sided structures illustrate the polysplenia syndrome. Classical findings in HS include cardiac malpositioning, septal defects, bilateral bilobed or trilobed lungs, midline liver, intestinal malrotation, and abnormal spleen development. Intestinal malrotation can lead to gut volvulus and ischemia^(5,6)^, whereas complete asplenia predisposes to bacterial infections and sepsis^(1,2)^. Up to 75% of patients with polysplenia have significant cardiac malformations, namely endocardial cushion defects, double-outlet right ventricle, left heart obstruction, and anomalous venous return^(4)^. The severity of congenital heart disease remains a main determinant of the long-term prognosis of HS patients—even after surgical repair of congenital heart disease, patients are prone to developing arrhythmias, thromboembolism due to right atrium enlargement^(7)^, and progressive systolic dysfunction^(3)^.

In conclusion, HS is a complex syndrome that has remarkable phenotypic variability and is a challenge to manage. Patients with HS are prone to develop potentially life-threatening complications, which should be promptly diagnosed and managed. Therefore, imaging studies are critical in evaluating these patients, because they delineate the spectrum of possible cardiac and extracardiac involvement in HS and associated complications.
